# Relationship between mortality and molecular epidemiology of methicillin-resistant *Staphylococcus aureus* bacteremia

**DOI:** 10.1371/journal.pone.0271115

**Published:** 2022-07-08

**Authors:** Masaru Shimizu, Toshihito Mihara, Junya Ohara, Keita Inoue, Mao Kinoshita, Teiji Sawa

**Affiliations:** 1 Department of Anesthesiology, University Hospital, Kyoto Prefectural University of Medicine, Kyoto, Japan; 2 Department of Anesthesia and Perioperative Care, University of California San Francisco, San Francisco, California, United States of America; 3 Department of Anesthesiology, Red Cross Kyoto Daiichi Hospital, Kyoto, Japan; Tribhuvan University, NEPAL

## Abstract

*Staphylococcus aureus* is the primary cause of bacteremia, and methicillin-resistant *S*. *aureus* bacteremia is associated with a high mortality rate. Methicillin-resistant *S*. *aureus* clones are widespread worldwide, and molecular epidemiological studies are important. Therefore, this study aimed to determine the characteristics of patients who died due to methicillin-resistant *S*. *aureus* bacteremia and microbiological characteristics of methicillin-resistant *S*. *aureus* strains in a tertiary teaching hospital. This single-center, retrospective study included patients with methicillin-resistant *S*. *aureus* isolated from blood bacterial culture performed at Kyoto Prefectural University of Medicine Hospital, from October 2016 to May 2019. The data analyzed included patient background, clinical strain characteristics, and molecular epidemiology. Of 41 patients with methicillin-resistant *S*. *aureus* bacteremia (median age, 60 [28–70] years; 24 (59%) were men), and 7 (17%) died due to methicillin-resistant *S*. *aureus* bacteremia. The median age of those who died in the methicillin-resistant *S*. *aureus* bacteremia group was predominantly higher than that of those in the alive group (p = 0.03). The most common cause of methicillin-resistant *S*. *aureus* bacteremia was endovascular devices, which occurred in 20 (49%), 18 (53%), and 2 (29%) patients in the total, alive, and died groups, respectively. Bacteriological characteristics showed that type IV Staphylococcal Cassette Chromosome *mec* genotype was most frequently detected in the total (n = 34 [83%]), alive (n = 29 [85%]), and died (n = 5 [71%]) groups. In the molecular cluster analysis, CC8, ST8, staphylococcal Cassette Chromosome *mec* type IV, and community-acquired-methicillin-resistant *S*. *aureus* formed the largest groups. The diversity of methicillin-resistant *S*. *aureus* clones is evident, and it is possible that clones with new virulence factors may still emerge. In the future, it will be crucial to monitor the epidemiological trends of methicillin-resistant *S*. *aureus* to respond quickly to changes in pathogenic and clonal factors, to clarify the gene expression network by identifying old and new virulence factors.

## Introduction

*Staphylococcus aureus*, a major cause of bacteremia in developed countries and a common cause of community-acquired (CA) and healthcare-associated (HA) bloodstream infections, has an incidence of 20–30 cases per 100,000 population per year in high-income countries [1]. Methicillin-resistant *S*. *aureus* (MRSA) is widely recognized as one of the most common drug-resistant pathogens causing hospital- and community-acquired infections.

Infections caused by drug-resistant bacteria result in worse outcomes [2]. Although efforts have continued to evolve in preventing MRSA infections [3], it remains a major cause of increased mortality and morbidity [4,5]. In particular, patients with both MRSA bacteremia and infective endocarditis have a high mortality rate of 17%–50% [6–9].

Currently, international MRSA clones, such as ST8-Staphylococcal Cassette Chromosome (SCC) *mec* IV (USA300 clone), ST1-SCC*mec* IV (USA400 clone), ST30-SCC*mec* IV (Southwest Pacific clone), ST59-SCC*mec* V (Taiwan clone), and ST80-SCC*mec* IV (European clone), are widespread worldwide [10,11]. Furthermore, HA-MRSA strains spread to the community, and CA-MRSA strains cause outbreaks in hospitals [12]. This epidemiological change is a major threat to public health. Therefore, detailed molecular epidemiological characterization, as suggested, could provide important information for combating MRSA infections, as well as for monitoring its trends and epidemiological pattern in Japanese hospitals [13]. Thus, the number of deaths due to MRSA bloodstream infections in Japan decreased from 5,924 in 2011 to 4,224 in 2017 [14]. We know from current surveillance and molecular epidemiological studies that specific clones of MRSA are closely associated with virulence factors and drug susceptibility and that these trends are important as a basis for infectious disease care, treatment, and control.

The purpose of this study was to determine the background characteristics of patients who died from MRSA bacteremia and microbiological characteristics of MRSA clinical strains from 2016 to 2019. We also conducted molecular epidemiological analysis based on various DNA sequences, such as SCC*mec*, multilocus sequence typing (MLST), and PCR-based ORF Typing (POT), to characterize MRSA bacteremia at a tertiary teaching hospital.

## Materials and methods

### MRSA isolation, storage, and culture

Between October 2016 and May 2019, the first clinical isolates of MRSA were obtained from blood bacterial cultures of patients whose physicians deemed blood bacterial culture tests to be necessary when they visited the emergency department or were admitted to a hospital ward. Blood bacterial cultures performed at the Department of Clinical Center, University Hospital, Kyoto Prefectural University of Medicine. Duplicate strains were excluded. Strains were stored at -80°C. Twenty-four hours prior to drug susceptibility testing, antibiotic susceptibility of *S*. *aureus*, SCC*mec* typing, MLST, POT type, and pathogen analysis, strains stored at -80°C were incubated in Tryptic Soy agar plates at 37°C for 18 h.

### Collection of patient data

This retrospective cohort study, conducted at the Kyoto Prefectural University of Medicine Hospital in Japan, was approved by the Medical Ethics Committee (approval number ERB-C-1174-2). Informed consent for publication of this study was obtained via an opt-out form on the website. We examined the medical records of patients to obtain information on age, sex, presence of MRSA carriage, history of surgery, Charlson Comorbidity Index [15], source site of MRSA bacteremia, location where specimens were collected, time to administration of susceptible and appropriate antimicrobial agents, and use of antibiotics in the past 30 days. There were no exclusion criteria.

### Details of this clinical study

This study was a clinical study of retrospective study. We used only medical information, without any medical invasion and intervention on the patients. Therefore, we used opt-out consent from patients. We published information on the website of the Department of Anesthesiology of Kyoto Prefectural University of Medicine about the purpose and conduct of the study, and further guaranteed that patients had the opportunity to refuse. The opt-out web address for this clinical study is https://anesth-kpum.org/research/mrsa%e6%84%9f%e6%9f%93%e7%97%87%e3%81%ae%e7%96%ab%e5%ad%a6%e3%81%ae%e5%a4%89%e9%82%84%e3%81%a8%e6%96%b0%e3%81%9f%e3%81%aa%e6%b2%bb%e7%99%82%e6%b3%95%e3%81%ae%e9%96%8b%e7%99%ba/.

### MRSA bacteremia and MRSA bacteremia-related death definition

MRSA bacteremia was defined as the presence of one or more positive blood cultures from a patient with clinical symptoms of infection, such as sweats, chills, and fever. MRSA bacteremia-related death was defined if the cause of death was an acute complication (septic shock, disseminated intravascular coagulation, acute lung injury) related to MRSA bacteremia, endocarditis (complications of heart failure due to endocarditis), or both underlying disease and MRSA bacteremia.

### DNA extraction

In this study, target strain DNA was extracted from isolates using the CicaGeneus® DNA Extraction Reagent Kit (Kanto Chemical, Tokyo, Japan) according to the manufacturer’s recommendations. This template DNA was used for all analyses.

### Antibiotic susceptibility of *S*. *aureus*

Antibiotic sensitivity was determined using the minimum inhibitory concentration (MIC). The MICs complied with the Clinical and Laboratory Standards Institute (CLSI) [16]. The breakpoints of resistance to each antibiotic were as follows: ampicillin (ABPC) ≥0.5 μg/mL, penicillin (PC) ≥0.25/mL, cefazolin (CEZ) ≥8/mL, gentamicin (GM) ≥16 μg/mL, amikacin (AMK) ≥64 μg/mL, erythromycin (EM) ≥8/mL, clindamycin (CLDM) ≥4 μg/mL, minocycline (MINO) ≥16/mL, vancomycin (VCM) ≥16 μg/mL, teicoplanin (TEIC) ≥32 μg/mL, ciprofloxacin (CPFX) ≥4 μg/mL, sulfamethoxazole (ST) ≥512 μg/mL, and linezolid (LZD) ≥4 μg/mL. The breakpoint of arbekacin was not defined by the CLSI; therefore, GM was used instead.

### SCC*mec* typing

Eight SCC*mec* typing synthesized primers were used in a previously reported multiplex polymerase chain reaction (PCR) method [17]. We determined SCC*mec* types-I (415 bp), II (937 bp), III (518 bp), IV (937 and 415 bp), and V (518 and 359 bp) targeting the genes *ccrA2-B*, *ccrC*, *IS1272*, and *mec*A-IS431. SCC*mec* types I, II, and III were defined as HA-MRSA, while types IV and V were defined as CA-MRSA [18].

### MLST analysis

In MLST, we created primers specific for each of the following genes to amplify seven housekeeping genes required for *S*. *aureus* survival: carbamate kinase (*arc*, 456 bp), shikimic acid dehydrogenase (*aroE*, 456 bp), glycerol kinase (*glpF*, 465 bp), guanylate kinase (*gmk*, 429 bp), phosphate acetyltransferase nucleotide sequence (*pta*, 474 bp), triose phosphate isomerase (*tpi*, 402 bp), and acetyl coenzyme A acetyltransferase (*yqiL*, 516 bp) [19]. We analyzed the allele profiles and determined the sequence types (STs) using a database (http://www.mlst.net).

### POT type analysis

We performed POT analysis for all MRSA isolates based on the Cica Geneus® Staph POT KIT (Kanto Chemical Co., Inc., Tokyo, Japan). Two sets of multiplex PCRs were performed according to the manufacturer’s instructions. The presence of 23 open reading frames (ORFs) was determined from agarose electrophoresis images and the POT type. The POT score resulted in three POT numbers: POT1, SCC*mec* element region; POT2, prophage-based ORF; and POT3, prophage-based ORF [20].

### Pathogen

We analyzed the expression of the gene encoding Panton-Valentine leukocidin (*lukF*-*PV*) by PCR [21].

### Data analysis

The normality of the distribution of continuous variables was tested using the Kolmogorov-Smirnov method. Variables with a normal distribution are presented as means and standard deviations, and variables with a non-normal distribution are presented as medians (interquartile ranges) and were compared using the Mann–Whitney U test. Categorical variables are shown as numbers (%), and the difference between the alive and died groups was tested using Fisher’s exact test. The significance of the relationships was determined using Spearman’s rank correlation coefficient. The test was two-tailed. The significance level was set at α<0.05. We did not calculate the statistical sample size. However, using the reported mortality rate of MRSA bacteremia as 34% [8] and the mortality rate of MRSA bacteremia in this study as 17%, the posterior power was 0.66 using a two-sided significance level of p<0.05. To identify clusters of patients with MRSA bacteremia, we performed a hierarchical clustering method using the seven housekeeping gene numbers of MLST, as well as POT-1, -2, and -3 numbers. The analysis was performed using Ward’s method with Euclidean square distance. EZR software version 1.41 (Saitama Medical Center, Jichi Medical University, Saitama, Japan) was used for all statistical analyses.

## Results

### Patient background characteristics

**Fig 1:**
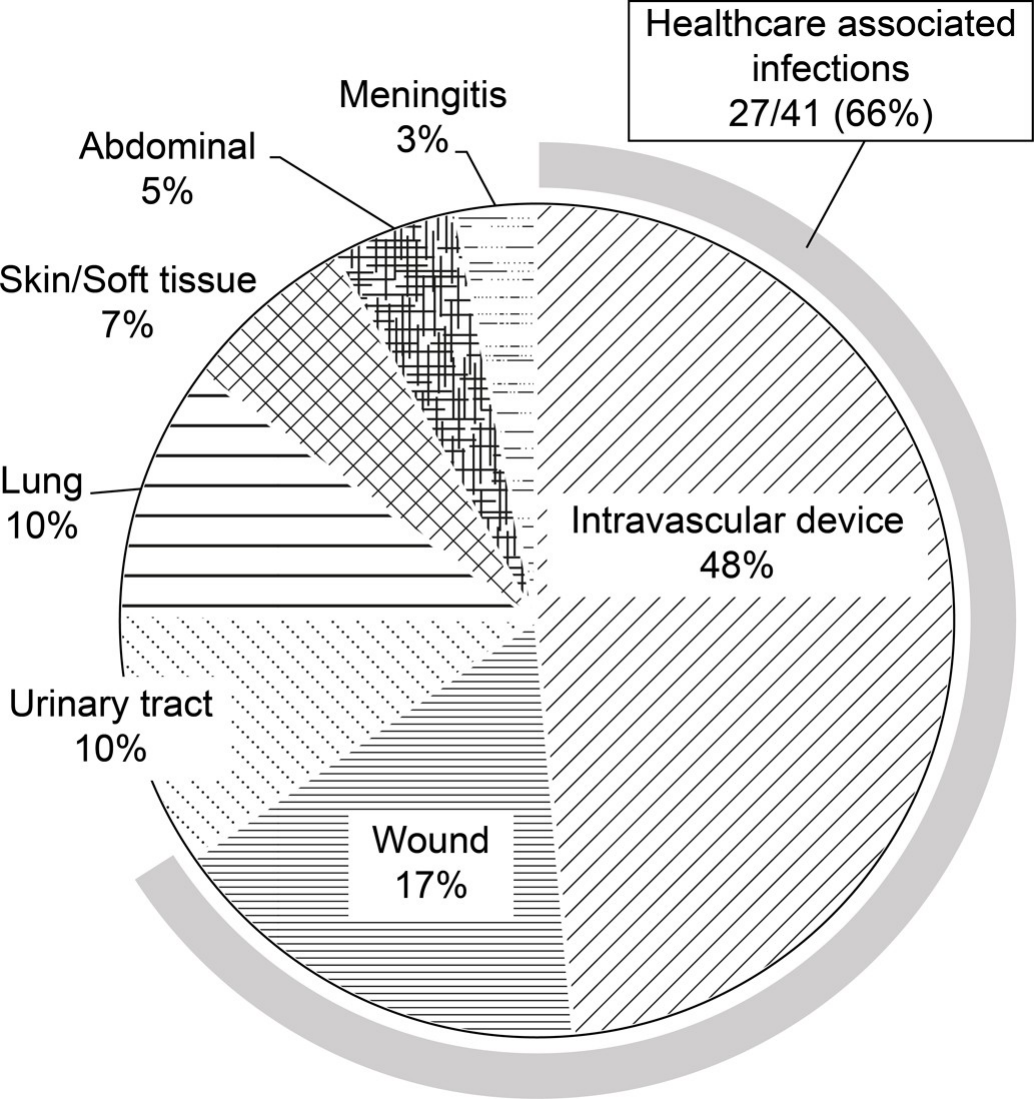
Distribution of the source of methicillin-resistant *Staphylococcus* aureus bloodstream infections.

MRSA strains were isolated from 41 bacteremia patients. Seven patients (17%) died due to MRSA bacteremia. The characteristics of patients who were alive or had died of MRSA bacteremia are shown in Table 1. The median age of those in the dead group (66 years) was significantly higher than that in the alive group (55.5 years, p = 0.03). There were no significant differences in sex, MRSA carriage, or previous surgery between the two groups. Differences in the Charlson comorbidity score and time to appropriate antimicrobial administration between groups were not statistically significant. The most common source of MRSA bacteremia was intravascular devices in the overall, alive, and dead groups (20/41 [49%], 18/34 [53%] and 2/7 [29%], respectively; Table 1), and HA infection (HAI) occurred in 27 (66%) patients (Fig 1). The most common place for blood culture collection was the general ward for the overall, alive, and dead groups (n = 23, n = 18 [53%], and n = 5 [71%]; respectively). There was no significant difference in treatment (intensive care unit treatment and antimicrobial exposure within 30 days) between the two groups. The time to onset of appropriate antimicrobial therapy tended to be earlier in the alive group (alive 30.5 [6.8–72.3] vs. dead 70 [40.3–114]).

**Table 1:** Patient background characteristics.

	Total patients (n = 41)	Alive (n = 34)	Dead (n = 7)	p value
**Characteristics**				
Age, years	60 [28–70]	55.5 [21–68]	66 [65–73]	0.03
**Sex, n (%)**				
Male	24 (59%)	20 (59%)	4 (57%)	1
Female	17 (41%)	14 (41%)	3 (43%)	1
MRSA career	20 (49%)	16 (47%)	4 (57%)	0.7
Surgical history	22 (54%)	18 (53%)	4 (57%)	1
Charlson Comorbidity Index	2 [1–4]	2 [1–4]	4 [2.5–6]	0.08
**Source of BSI**				
Intravascular device	20 (49%)	18 (53%)	2 (29%)	0.22
Wound	7 (17%)	5 (15%)	2 (29%)	
Urinary tract	4 (10%)	3 (9%)	1 (14%)	
Lung	4 (10%)	4 (12%)	0	
Skin/Soft tissue	3 (7%)	3 (9%)	0	
Abdominal	2 (5%)	1 (3%)	1 (14%)	
Meningitis	1 (3%)	0	1 (14%)	
**Detected location information**				
General ward	23 (56%)	18 (53%)	5 (71%)	0.46
ICU	7 (17%)	7 (21%)	0	
Outpatient	11 (27%)	9 (26%)	2 (29%)	
**Clinical measure**				
Time to appropriate antimicrobial therapy (h)	30.5 [6.8–72.3]	25 [4.3–68]	70 [40.3–114]	0.15
ICU care	12 (29%)	11(32%)	1(14%)	0.65
Antimicrobial exposure within 30 days	19 (46%)	17 (50%)	2 (29%)	0.42

Demographics and characteristics of mortality after 30 days in patients with MRSA bacteremia at the University Hospital of the Kyoto Prefectural University of Medicine from October 2016 to May 2019.

Data presented as medians [IQRs] or n (%).

MRSA, methicillin-resistant *Staphylococcus* aureus; BSI, bloodstream infection; ICU, intensive care unit.

### Microbiological characteristics of MRSA

The most isolated genotype of SCC*mec* was type IV (n = 34, 83%) and type II (n = 5, 12%). There were no significant differences in SCC*mec* genotypes I to V between the two groups (p = 0.34). Bacteriological CA-MRSA accounted for 85% (n = 35) of the total cases. There was no expression of *lukF*-*PV* in any of the strains. The strains were resistant to the following antibiotics: ABPC, PC, and CEZ (41 strains, 100%); CPFX (35 strains, 85%); EM (34 strains, 83%); and tetracycline (11 strains, 27%). Resistance to tetracycline was significantly higher in the dead group than in the alive group (alive n = 8 [23%] vs. died n = 3 [43%], p = 0.04) (Table 2). All strains were sensitive to AMK, VCM, TEIC, ST, and LZD.

**Table 2:** Microbiological characteristics of MRSA.

	Total patients (n = 41)	Alive (n = 34)	Dead (n = 7)	p value
**Infection classification**				
**SCC*mec* type**				
**Ⅰ**	1 (2%)	0	1 (14%)	0.34
**Ⅱ**	5 (12%)	4 (12%)	1 (14%)	
**Ⅲ**	0	0	0	
**Ⅳ**	34 (83%)	29 (85%)	5 (71%)	
**Ⅴ**	1 (2%)	1 (3%)	0	
**CA-MRSA**	35 (85%)	30 (88%)	5 (71%)	0.58
**HA-MRSA**	6 (15%)	4 (12%)	2 (29%)	
**Antibiotic susceptibility profile**				
**GM (MIC ≥16 μg/mL)**	21 (51%)	16 (47%)	5 (71%)	0.6
**EM (MIC ≥8 μg/mL)**	34 (83%)	28 (82%)	6 (86%)	1
**CLDM (MIC ≥4 μg/mL)**	15 (37%)	14 (41%)	1 (14%)	0.12
**MINO (MIC ≥16 μg/mL)**	11 (27%)	8 (23%)	3 (43%)	0.04
**CPFX (MIC ≥8 μg/mL)**	35 (85%)	29 (85%)	6 (86%)	1
**LZD (MIC ≥8)**	0	0	0	
**VCM (MIC ≥16)**	0	0	0	

Data presented as n (%).

MRSA, methicillin-resistant *Staphylococcus aureus*; SCC, Staphylococcal cassette chromosome; CA-MRSA, community-acquired MRSA; HA-MRSA, healthcare-acquired MRSA; MIC, minimum inhibitory concentration; GM, gentamicin; EM, erythromycin; CLDM, clindamycin; MINO, minocycline; CPFX, ciprofloxacin; LZD, linezolid; VCM, vancomycin.

### Clone determination by MLST

MLST analysis showed that the most common sequence type was ST8 (n = 25, 61%), followed by ST764 (n = 5, 12%). The most common clone complex (CC) was CC8 (n = 25, 61%), followed by CC1 (n = 8, 20%) and CC5 (n = 7, 17%). Six patients died of CC8 (85%). In addition, there was one new strain for which ST and CC could not be detected in the MLST database.

### Analysis by POT method

**Fig 2:**
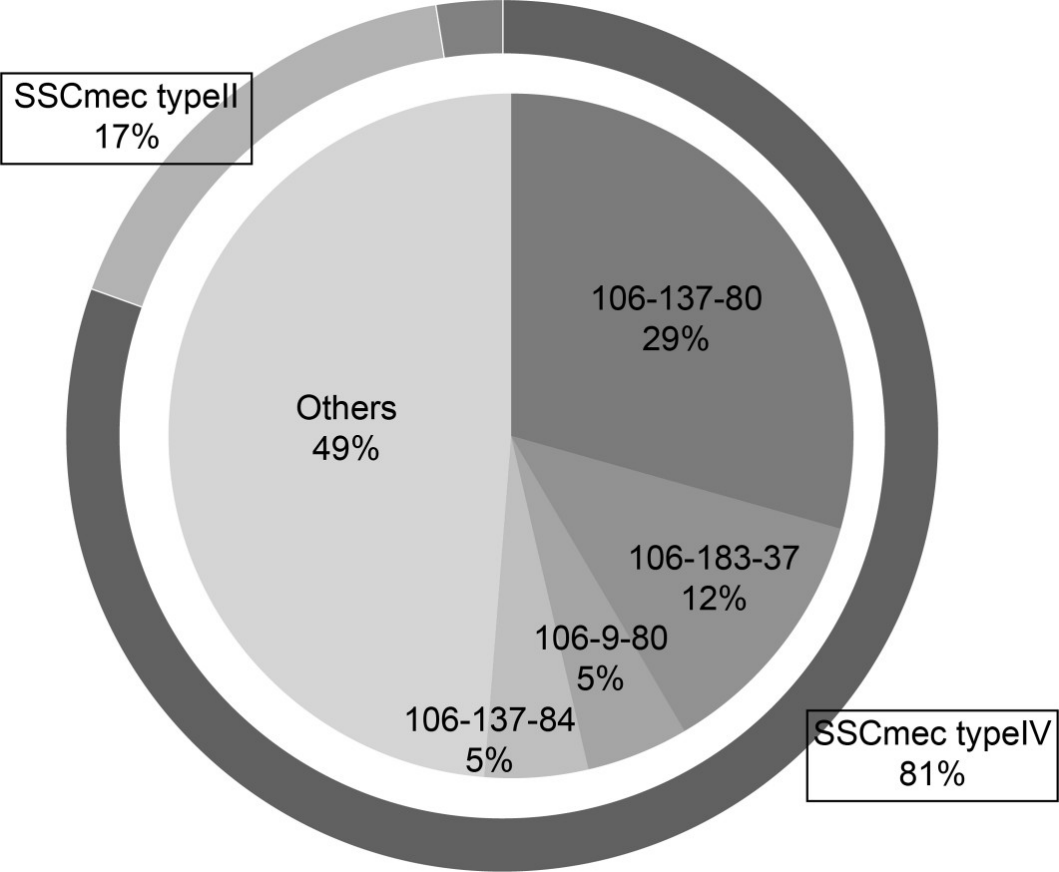
Distribution of Staphylococcal Cassette Chromosome *mec* genotypes and PCR-based ORF Typing methods.

Twenty-six different POT types were detected, with four types identified multiple times; the most frequently isolated POT type was 106-137-80 with 12 strains (29%), followed by 106-183-37 with 5 strains (12%) (Fig 2). Some strains with the same POT type and antimicrobial resistance pattern were detected. However, it was not HAI because the patients were admitted during a different hospitalization period.

### Cluster analysis

**Fig 3:**
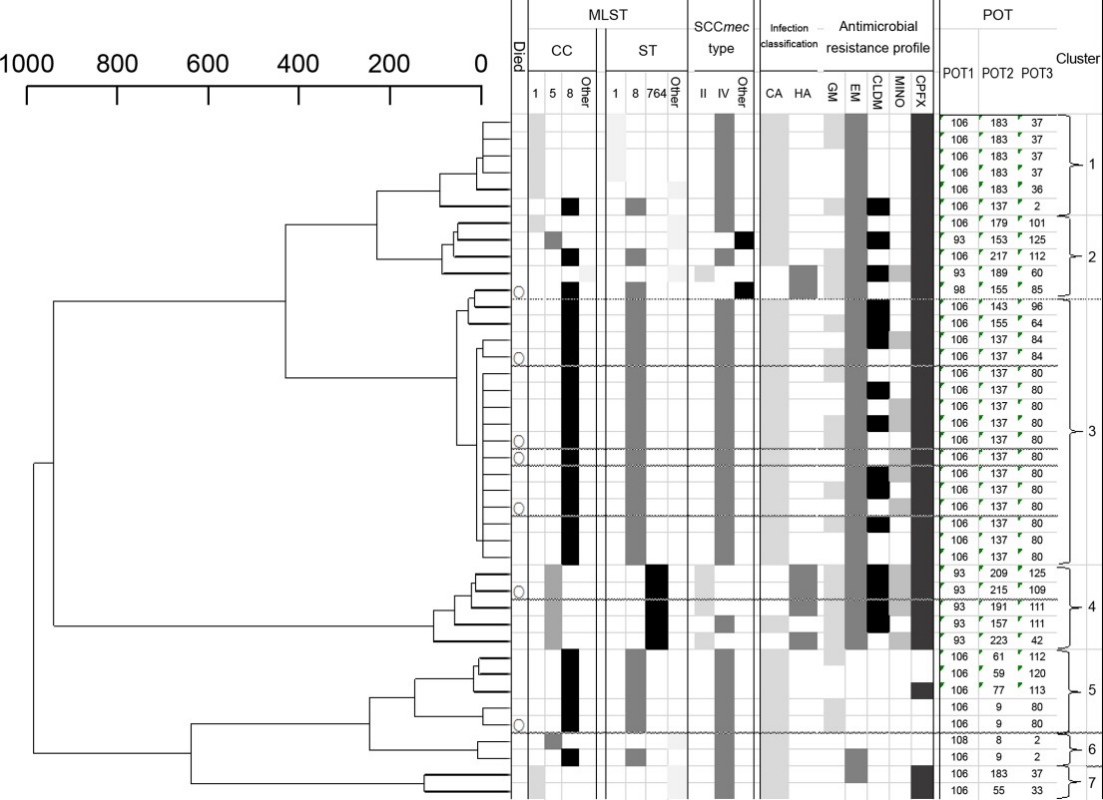
Tree diagram and the results of the molecular epidemiological analysis of methicillin-resistant *Staphylococcus aureus* clones. First row: Died within 30 days due to MRSA bacteremia. Second row: Clone complex relationship analyzed by MLST. Third row: Sequence-type relationships analyzed by MLST. Fourth row: SCC*mec* typing classification. Fifth row: Classification of bacteriology. Sixth row: Antimicrobial resistance patterns. Seventh row: POT number. Eighth row: Cluster number.

The results of the MLST and POT type analyses showed seven clusters of MRSA clinical strains with a dissimilarity of 200 (Fig 3). Cluster 3 was the largest, with 17 strains (41%). All strains in cluster 3 except for one and all strains in cluster 5 were CC8, ST8, SCCmec type IV, and CA. All strains in cluster 3 were resistant to EM, while all strains in cluster 5 were susceptible to EM. All strains in clusters 5, 6, and 7 were sensitive to CLDM and MINO. The proportion of CA and HA in each cluster was higher in cluster 4 (80%) than in the other clusters, with four HA strains in cluster 4.

## Discussion

This is one of the few studies on MRSA bacteremia in designated medical institutions for Class 1 infectious diseases in Japan. The main findings of this current study are as follows: dead patients with MRSA bacteremia were significantly older and had a 2.8 times longer time to administration of susceptible and appropriate antimicrobial agents than those in the alive group. Approximately 50% of MRSA bacteremia occurred among those with intravascular devices. The microbiological characteristics of the MRSA clinical strains demonstrated that high resistance occurred more with MINO in the dead group compared with the alive group. Molecular immunology analysis suggested that 83% of all MRSA strains were SCCmec type IV, with ST8 and CC8 being the most common. Surprisingly, we observed that 85% of the pathogenic bacteria in hospital-acquired infections were bacteriological CA-MRSA.

The mortality rate due to *S*. *aureus* bacteremia is 20%–30%, the mortality rate due to MRSA bacteremia is even higher (20%–50%), and the cure rate for MRSA infections is 50%–60% [22–24]. In our study, the mortality rate due to MRSA bacteremia was 17%. The risk factors for mortality in MRSA bacteremia are age, catheter device use, and exposure to macrolides [25–27]. Our study showed that those who died were older than those alive. The number of deaths due to drug-resistant pathogens was reported to be 700,000 per year worldwide [28], including 33,110 in Europe [29], >35,000 in the United States [30], and approximately 8,000 in Japan [31]. In addition, it is estimated to be even more serious in developing countries [28]. Early administration of appropriate antimicrobial agents that are effective against MRSA implies, in other words, early diagnosis and early therapy. In this study, the time taken to administer appropriate antimicrobial agents was 2.8 times longer in the dead group than in the alive group. A delay in the administration of appropriate antimicrobial agents could have significant harmful effects on treatment. In particular, patients with weak resistance and severe diseases, such as sepsis, cause increased medical costs due to higher mortality rates and longer hospital stays [32]. In Japan, the burden due to MRSA and drug-resistant *Escherichia coli* is high because of healthcare cost, and the burden due to MRSA is 3.6 times higher than that in Europe [33]. However, using current diagnostic methods, it is difficult to accurately diagnose infections caused by resistant bacteria, often delaying the initiation of appropriate treatment [34]. Therefore, it is important to initiate appropriate antimicrobial therapy at an early stage for a more effective treatment. The most common source of infection for MRSA bacteremia is intravascular device infection, at approximately 30% [35,36]. In this study, intravascular device infections were the most common, at approximately 50%. Early removal of devices in MRSA bacteremia is important because MRSA forms biofilms, which reduce the effectiveness of antimicrobial agents [37,38]. In addition, catheter-related bacteremia is strongly associated with in-hospital mortality [27].

The microbiological characteristics of MRSA revealed greater resistance against MINO among those in the dead group than among those in the alive group. Worldwide, the overall use of antimicrobial agents has increased by 65% over the past 16 years [39]. In Japan, the use of antimicrobial agents, including tetracyclines, has increased. We expected this increased antimicrobial resistance because tetracycline is also widely used as a topical drug in hospitals. Molecular epidemiological characteristics in this study showed that 83% of all MRSA strains were SCC*mec* type IV. In the past, type II, frequently found in HA-MRSA, accounted for approximately 75% of cases [40]. At that time, HAI caused by CA-MRSA was a serious problem in European countries and the United States. Surprisingly, this study revealed an increase in HAI due to CA-MRSA, which accounted for 85% of all MRSA strains. We suggest that the genetic background of MRSA has changed significantly because various CA-MRSA strains entered the hospital to compete with HA-MRSA for survival. The factors influencing this entry include the increased carrier rate of MRSA and easy transmission between MRSA strains. The carrier rates of MRSA and multidrug-resistant gram-negative bacteria are higher in nursing homes and healthcare workers with a long-term work history [41–43]. The MRSA carrier rate in Japan is 31.4% [44], and it was as high as approximately 50% in this study. Therefore, it is easy to contract MRSA in daily life with an easy spread between strains. In addition, 1% of the patients admitted to the intensive care unit are new carriers of MRSA [45]. We consider that HA-MRSA came into the community, and CA-MRSA, which was highly susceptible to antimicrobial agents, developed multidrug resistance, as well as HA-MRSA.

In this study, CC8 and ST8 were most prevalent in the MLST analysis. The major representative of CA-MRSA is the CC8 clone (USA300) [18]. USA300 is a CC8, ST8, and *lukF-PV* gene-positive strain that increased dramatically in the United States in the first half of 2000 [46,47] and was first reported in Japan in 2007 [48].

In this study, all MRSA strains were *lukF-PV* gene-negative, and we concluded that some were the Japanese-intrinsic CA-MRSA (CA-MRSA/J) genetically similar to the USA300 type [49].

In Japan, CA-MRSA/J has increased [50]. It includes the virulence factors, toxic shock syndrome toxin-1, and enterotoxin and is reported to be potentially highly virulent and severe, with high expression of toxic shock syndrome toxin-1 [49,51]. In addition, in recent years, the existence or absence of the *lukF-PV* gene has made no difference in virulence; thus, we suggest that there are pathogenic factors other than the *lukF-PV* gene [52]. High drug resistance was strongly associated with the virulence factors of *S*. *aureus* [53]. The POT index –106-137-80 was the most frequently isolated and is the most frequently isolated and commonly reported in hospitals in Japan [54,55]. Although the POT index cannot be used to estimate the existence of the *lukF-PV* gene and other virulence factors, it is useful for infection control by monitoring antibiotic susceptibility and the course of the disease. The POT method detects ORFs, which are the mobile regions of the DNA chromosomes. Therefore, the POT index can be altered by genetic mutations [56–58]. Furthermore, the POT method is a quick and simple test, although it should be used for comprehensive evaluation.

In this study, we combined MLST analysis with the POT method for cluster analysis to improve the resolution of MRSA; in groups 5, 6, and 7, in contrast to other groups, the antibiogram showed CLDM and MINO antimicrobial susceptibility. CA-MRSA is highly susceptible to CLDM, MINO, quinolones, and aminoglycoside [11]. However, in this study, CA-MRSA accounted for 85% of the total cases, with reduced susceptibility to many types of antibiotic agents.

There are some limitations to our study; first, it is a review of retrospective studies of blood isolates at a single center only. Second, we classified the patients into two groups: alive and dead, although the data were unbalanced, and the sample size and analyzing power were small. In the future, it will be necessary to include multiple-center large-scale studies and verify the correlation by adding clinical analysis of blood data.

## Conclusions

To the best of our knowledge, our study is one of the few studies that have focused on understanding MRSA bacteremia. We have revealed the characteristics of MRSA in specific regions. The diversity of MRSA clones is remarkable, and it is possible that clones with new virulence factors will appear in the future. Therefore, it is very important to clarify the gene expression network by identifying old and new virulence factors and monitor the epidemiological trends of MRSA clones continuously and carefully and respond quickly to changes.

## References

[1] Laupland KB, Lyytikäinen O, Søgaard M, Kennedy KJ, Knudsen JD, Ostergaard C, et al. International Bacteremia Surveillance Collaborative. The changing epidemiology of Staphylococcus aureus bloodstream infection: a multinational population-based surveillance study. Clin Microbiol Infect. 2013;19(5): 465–471. doi: 10.1111/j.1469-0691.2012.03903.x 22616816

[2] Barrasa-Villar JI, Aibar-Remón C, Prieto-Andrés P, Mareca-Doñate R, Moliner-Lahoz J. Impact on morbidity, mortality, and length of stay of hospital-acquired infections by resistant microorganisms. Clin Infect Dis. 2017;65(4): 644–652. doi: 10.1093/cid/cix411 28472416

[3] Kallen AJ, Mu Y, Bulens S, Reingold A, Petit S, Gershman K, et al. Active Bacterial Core surveillance (ABCs) MRSA Investigators of the Emerging Infections Program. Health care-associated invasive MRSA infections, 2005–2008. JAMA. 2010;304(6): 641–648. doi: 10.1001/jama.2010.1115 20699455

[4] van Hal SJ, Jensen SO, Vaska VL, Espedido BA, Paterson DL, Gosbell IB. Predictors of mortality in Staphylococcus aureus bacteremia. Clin Microbiol Rev. 2012;25(2): 362–386. doi: 10.1128/CMR.05022-11 22491776 PMC3346297

[5] Pérez-Montarelo D, Viedma E, Larrosa N, Gómez-González C, Ruiz de Gopegui E, Muñoz-Gallego I, et al. Molecular epidemiology of *Staphylococcus aureus* bacteremia: association of molecular factors with the source of infection. Front Microbiol. 2018;9: 2210. doi: 10.3389/fmicb.2018.02210 30319561 PMC6167439

[6] Inagaki K, Lucar J, Blackshear C, Hobbs CV. Methicillin-susceptible and methicillin-resistant Staphylococcus aureus bacteremia: nationwide estimates of 30-day readmission, in-hospital mortality, length of stay, and cost in the United States. Clin Infect Dis. 2019;69(12): 2112–2118. doi: 10.1093/cid/ciz123 30753447

[7] Uematsu H, Yamashita K, Mizuno S, Kunisawa S, Shibayama K, Imanaka Y. Effect of methicillin-resistant *Staphylococcus aureus* in Japan. Am J Infect Control. 2018;46(10): 1142–1147. doi: 10.1016/j.ajic.2018.04.214 29784441

[8] Cosgrove SE, Sakoulas G, Perencevich EN, Schwaber MJ, Karchmer AW, Carmeli Y. Comparison of mortality associated with methicillin-resistant and methicillin-susceptible Staphylococcus aureus bacteremia: a meta-analysis. Clin Infect Dis. 2003;36(1): 53–59. doi: 10.1086/345476 12491202

[9] Gasch O, Camoez M, Dominguez MA, Padilla B, Pintado V, Almirante B, et al. REIPI/GEIH Study Groups. Predictive factors for mortality in patients with methicillin-resistant Staphylococcus aureus bloodstream infection: impact on outcome of host, microorganism and therapy. Clin Microbiol Infect. 2013;19(11): 1049–1057. doi: 10.1111/1469-0691.12108 23331461

[10] Stefani S, Chung DR, Lindsay JA, Friedrich AW, Kearns AM, Westh H, et al. Meticillin-resistant *Staphylococcus aureus* (MRSA): global epidemiology and harmonisation of typing methods. Int J Antimicrob Agents. 2012;39(4): 273–282. doi: 10.1016/j.ijantimicag.2011.09.030 22230333

[11] Lee AS, de Lencastre H, Garau J, Kluytmans J, Malhotra-Kumar S, Peschel A, et al. Methicillin-resistant Staphylococcus aureus. Nat Rev Dis Primers. 2018;4: 18033. doi: 10.1038/nrdp.2018.33 29849094

[12] David MZ, Glikman D, Crawford SE, Peng J, King KJ, Hostetler MA, et al. What is community-associated methicillin-resistant Staphylococcus aureus? J Infect Dis. 2008;197(9): 1235–1243. doi: 10.1086/533502 18422435

[13] Yang X, Zhao J, Wang Y, Wu J, Wang X, Wang Y, et al. Molecular epidemiology of methicillin-resistant *Staphylococcus aureus* in hospitalized patients in eastern Heilongjiang Province, China. Infect Drug Resist. 2021;14: 1635–1643. doi: 10.2147/IDR.S307856 33953574 PMC8089471

[14] Tsuzuki S, Matsunaga N, Yahara K, Gu Y, Hayakawa K, Hirabayashi A, et al. National trend of blood-stream infection attributable deaths caused by Staphylococcus aureus and Escherichia coli in Japan. J Infect Chemother. 2020;26(4): 367–371. doi: 10.1016/j.jiac.2019.10.017 31801696

[15] Charlson ME, Pompei P, Ales KL, MacKenzie CR. A new method of classifying prognostic comorbidity in longitudinal studies: development and validation. J Chronic Dis. 1987;40(5): 373–383. doi: 10.1016/0021-9681(87)90171-8 3558716

[16] CLSI.; Methods for dilution antimicrobial susceptibility tests for bacteria that grow aerobically; approved Standard-eighth edition M7-A8. Clinical and Laboratory Standards Institute. Wayne, PA, USA: Clinical and Laboratory Standards Institute, (2009).

[17] Boye K, Bartels MD, Andersen IS, Møller JA, Westh H. A new multiplex PCR for easy screening of methicillin-resistant *Staphylococcus aureus* SCCmec types I-V. Clin Microbiol Infect. 2007;13(7): 725–727. doi: 10.1111/j.1469-0691.2007.01720.x 17403127

[18] David MZ, Daum RS. Community-associated methicillin-resistant Staphylococcus aureus: epidemiology and clinical consequences of an emerging epidemic. Clin Microbiol Rev. 2010;23(3): 616–87. doi: 10.1128/CMR.00081-09 20610826 PMC2901661

[19] Enright MC, Day NP, Davies CE, Peacock SJ, Spratt BG. Multilocus sequence typing for characterization of methicillin-resistant and methicillin-susceptible clones of Staphylococcus aureus. J Clin Microbiol. 2000;38(3): 1008–1015. doi: 10.1128/JCM.38.3.1008-1015.2000 10698988 PMC86325

[20] Maeda T, Saga T, Miyazaki T, Kouyama Y, Harada S, Iwata M, et al. Genotyping of skin and soft tissue infection (SSTI)-associated methicillin-resistant Staphylococcus aureus (MRSA) strains among outpatients in a teaching hospital in Japan: application of a phage-open reading frame typing (POT) kit. J Infect Chemother. 2012;18(6): 906–914. doi: 10.1007/s10156-012-0506-4 23150115

[21] Stegger M, Andersen PS, Kearns A, Pichon B, Holmes MA, Edwards G, et al. Rapid detection, differentiation and typing of methicillin-resistant Staphylococcus aureus harbouring either mecA or the new mecA homologue mecA (LGA251). Clin Microbiol Infect. 2012;18(4): 395–400. doi: 10.1111/j.1469-0691.2011.03715.x 22429460

[22] Bayer AS, Lam K, Ginzton L, Norman DC, Chiu CY, Ward JI. Staphylococcus aureus bacteremia. Clinical, serologic, and echocardiographic findings in patients with and without endocarditis. Arch Intern Med. 1987;147(3): 457–462. doi: 10.1001/archinte.147.3.457 3103561

[23] Cosgrove SE, Sakoulas G, Perencevich EN, Schwaber MJ, Karchmer AW, Carmeli Y. Comparison of mortality associated with methicillin-resistant and methicillin-susceptible *Staphylococcus aureus* bacteremia: a meta-analysis. Clin Infect Dis. 2003;36(1): 53–59. doi: 10.1086/345476 12491202

[24] Song KH, Kim ES, Sin HY, Park KH, Jung SI, Yoon N, et al. Characteristics of invasive Staphylococcus aureus infections in three regions of Korea, 2009–2011: a multi-center cohort study. BMC Infect Dis. 2013;13: 581. doi: 10.1186/1471-2334-13-581 24321206 PMC4029571

[25] Cuervo G, Gasch O, Shaw E, Camoez M, Domínguez MÁ, Padilla B, et al. REIPI/GEIH study group. Clinical characteristics, treatment and outcomes of MRSA bacteraemia in the elderly. J Infect. 2016;72(3): 309–316. doi: 10.1016/j.jinf.2015.12.009 26723914

[26] Wi YM, Rhee JY, Kang CI, Chung DR, Song JH, Peck KR. Clinical predictors of methicillin-resistance and their impact on mortality associated with Staphylococcus aureus bacteraemia. Epidemiol Infect. 2018;146(10): 1326–1336. doi: 10.1017/S0950268818001255 29781425 PMC9134285

[27] Niek WK, Teh CSJ, Idris N, Sit PS, Lee YQ, Thong KL, et al. Methicillin-resistant Staphylococcus aureus bacteraemia, 2003–2015: comparative evaluation of changing trends in molecular epidemiology and clinical outcomes of infections. Infect Genet Evol. 2020;85: 104567. doi: 10.1016/j.meegid.2020.104567 32980576

[28] O’Neill J. Tackling drug-resistant infections globally: final report and recommendations. Review on Antimicrobial Resistance. May 2016.

[29] Cassini A, Högberg LD, Plachouras D, Quattrocchi A, Hoxha A, Simonsen GS, et al. Burden of AMR Collaborative Group. Attributable deaths and disability-adjusted life-years caused by infections with antibiotic-resistant bacteria in the EU and the European Economic Area in 2015: a population-level modelling analysis. Lancet Infect Dis. 2019;19(1): 56–66. doi: 10.1016/S1473-3099(18)30605-4 30409683 PMC6300481

[30] CDC. Antibiotic resistance threats in the United States 2019. Available from: https://www.cdc.gov/drugresistance/pdf/threats-report/2019-ar-threats-report-508.pdf. Accessed August 22, 2021.

[31] Tsuzuki S, Matsunaga N, Yahara K, Gu Y, Hayakawa K, Hirabayashi A, et al. National trend of blood-stream infection attributable deaths caused by Staphylococcus aureus and Escherichia coli in Japan. J Infect Chemother. 2020;26(4): 367–371. doi: 10.1016/j.jiac.2019.10.017 31801696

[32] Falcone M, Bassetti M, Tiseo G, Giordano C, Nencini E, Russo A, et al. Time to appropriate antibiotic therapy is a predictor of outcome in patients with bloodstream infection caused by KPC-producing *Klebsiella pneumoniae*. Crit Care. 2020;24(1): 29. doi: 10.1186/s13054-020-2742-9 32000834 PMC6993311

[33] Cassini A, Högberg LD, Plachouras D, Quattrocchi A, Hoxha A, Simonsen GS, et al. Burden of AMR Collaborative Group. Attributable deaths and disability-adjusted life-years caused by infections with antibiotic-resistant bacteria in the EU and the European Economic Area in 2015: a population-level modelling analysis. Lancet Infect Dis. 2019;19(1): 56–66. doi: 10.1016/S1473-3099(18)30605-4 30409683 PMC6300481

[34] Lodise TP, Berger A, Altincatal A, Wang R, Bhagnani T, Gillard P, et al. Antimicrobial resistance or delayed appropriate therapy-does one influence outcomes more than the other among patients with serious infections due to carbapenem-resistant versus carbapenem-susceptible enterobacteriaceae? Open Forum Infect Dis. 2019;6(6): ofz194. doi: 10.1093/ofid/ofz194 31198817 PMC6546203

[35] Alhunaif SA, Almansour S, Almutairi R, Alshammari S, Alkhonain L, Alalwan B, et al. Methicillin-resistant Staphylococcus aureus bacteremia: epidemiology, clinical characteristics, risk factors, and outcomes in a tertiary care center in Riyadh, Saudi Arabia. Cureus. 2021;13(5): e14934. doi: 10.7759/cureus.14934 34123631 PMC8189534

[36] Harbarth S, Rutschmann O, Sudre P, Pittet D. Impact of methicillin resistance on the outcome of patients with bacteremia caused by *Staphylococcus aureus*. Arch Intern Med. 1998;158(2): 182–189. doi: 10.1001/archinte.158.2.182 9448557

[37] Oyama T, Miyazaki M, Yoshimura M, Takata T, Ohjimi H, Jimi S. Biofilm-forming methicillin-resistant *Staphylococcus aureus* survive in Kupffer cells and exhibit high virulence in mice. 2016;8(7): 198. doi: 10.3390/toxins8070198 PMC4963831 27376326

[38] Miyazaki M, Takata T, Yoshimura H, Matsunaga A, Ohta D, Ishikura H, et al. Vancomycin bactericidal activity as a predictor of 30-day mortality in patients with methicillin-resistant *Staphylococcus aureus* bacteremia. Antimicrob Agents Chemother. 2011;55(4): 1819–1820. doi: 10.1128/AAC.01536-10 21282441 PMC3067189

[39] Klein EY, Van Boeckel TP, Martinez EM, Pant S, Gandra S, Levin SA, et al. Global increase and geographic convergence in antibiotic consumption between 2000 and 2015. Proc Natl Acad Sci U S A. 2018;115(15): E3463–E3470. doi: 10.1073/pnas.1717295115 29581252 PMC5899442

[40] Muraki Y, Yagi T, Tsuji Y, Nishimura N, Tanabe M, Niwa T, et al. Japanese antimicrobial consumption surveillance: first report on oral and parenteral antimicrobial consumption in Japan (2009–2013). J Glob Antimicrob Resist. 2016;7: 19–23. doi: 10.1016/j.jgar.2016.07.002 27973324

[41] Shih HI, Chang CM, Shen FC, Lee YJ, Wu CH, Hsu HC, et al. High prevalence nasal carriage of methicillin-resistant Staphylococcus aureus among long term care facility healthcare workers in relation to patient contact. Infect Prev Pract. 2021;3(1): 100117. doi: 10.1016/j.infpip.2021.100117 34368736 PMC8336196

[42] Wu TH, Lee CY, Yang HJ, Fang YP, Chang YF, Tzeng SL, et al. Prevalence and molecular characteristics of methicillin-resistant Staphylococcus aureus among nasal carriage strains isolated from emergency department patients and healthcare workers in central Taiwan. J Microbiol Immunol Infect. 2019;52(2): 248–254. doi: 10.1016/j.jmii.2018.08.015 30292763

[43] Liu H, Fei CN, Zhang Y, Liu GW, Liu J, Dong J. Presence, distribution and molecular epidemiology of multi-drug-resistant Gram-negative bacilli from medical personnel of intensive care units in Tianjin, China, 2007–2015. J Hosp Infect. 2017;96(2): 101–110. doi: 10.1016/j.jhin.2017.01.012 28268024

[44] Wakatake H, Fujitani S, Kodama T, Kawamoto E, Yamada H, Yanai M, et al. Positive clinical risk factors predict a high rate of methicillin-resistant *Staphylococcus aureus* colonization in emergency department patients. Am J Infect Control. 2012;40(10): 988–991. doi: 10.1016/j.ajic.2012.01.017 22627097

[45] Alfouzan W, Dhar R, Udo E. Genetic lineages of methicillin-resistant *Staphylococcus aureus* acquired during admission to an intensive care unit of a general hospital. Med Princ Pract. 2017;26(2): 113–117. doi: 10.1159/000453268 27829243 PMC5588361

[46] David MZ, Glikman D, Crawford SE, Peng J, King KJ, Hostetler MA, et al. What is community-associated methicillin-resistant Staphylococcus aureus?. J Infect Dis. 2008;197(9): 1235–1243. doi: 10.1086/533502 18422435

[47] Liu C, Graber CJ, Karr M, Diep BA, Basuino L, Schwartz BS, et al. A population-based study of the incidence and molecular epidemiology of methicillin-resistant *Staphylococcus aureus* disease in San Francisco, 2004–2005. Clin Infect Dis. 2008;46(11): 1637–1646. doi: 10.1086/587893 18433335

[48] Shibuya Y, Hara M, Higuchi W, Takano T, Iwao Y, Yamamoto T. Emergence of the community-acquired methicillin-resistant Staphylococcus aureus USA300 clone in Japan. J Infect Chemother. 2008;14(6): 439–441. doi: 10.1007/s10156-008-0640-1 19089559

[49] Iwao Y, Ishii R, Tomita Y, Shibuya Y, Takano T, Hung WC, et al. The emerging ST8 methicillin-resistant Staphylococcus aureus clone in the community in Japan: associated infections, genetic diversity, and comparative genomics. J Infect Chemother. 2012;18(2): 228–240. doi: 10.1007/s10156-012-0379-6 22350401

[50] Sasai N, Nakaminami H, Iwasaki M, Iwao M, Misegawa K, Hasui M, et al. Clonal change of methicillin-resistant *Staphylococcus aureus* isolated from patients with impetigo in Kagawa, Japan. J Dermatol. 2019;46(4): 301–307. doi: 10.1111/1346-8138.14820 30803017

[51] Kitagawa H, Ohge H, Hisatsune J, Kajihara T, Katayama K, Takahashi S, et al. Prosthetic valve endocarditis caused by ST8 SCCmecIVl type community-associated methicillin-resistant *Staphylococcus aureus*. Intern Med. 2019;58(5): 743–747. doi: 10.2169/internalmedicine.1415-18 30333402 PMC6443554

[52] Voyich JM, Otto M, Mathema B, Braughton KR, Whitney AR, Welty D, et al. Is Panton-Valentine leukocidin the major virulence determinant in community-associated methicillin-resistant *Staphylococcus aureus* disease? J Infect Dis. 2006;194(12): 1761–1770. doi: 10.1086/509506 17109350

[53] Kim HJ, Choi Q, Kwon GC, Koo SH. Molecular epidemiology and virulence factors of methicillin-resistant *Staphylococcus aureus* isolated from patients with bacteremia. J Clin Lab Anal. 2020;34(3): e23077. doi: 10.1002/jcla.23077 31721291 PMC7083439

[54] Takahashi H, Seki M, Yamamoto N, Hamaguchi S, Ojima M, Hirose T, et al. Validation of a phage-open reading frame typing kit for rapid identification of methicillin-resistant *Staphylococcus aureus* (MRSA) transmission in a tertiary hospital. Infect Drug Resist. 2015;8: 107–111. doi: 10.2147/IDR.S83509 25999746 PMC4437523

[55] Nakaie K, Yamada K, Park K, Nakamura Y, Okada Y, Fujita A, et al. Effectiveness of weekly polymerase chain reaction-based open reading frame typing analysis of all newly isolated methicillin-resistant *Staphylococcus aureus* strains for controlling nosocomial infections. J Infect Chemother. 2016;22(11): 733–737. doi: 10.1016/j.jiac.2016.07.007 27693014

[56] O’Sullivan MV, Kong F, Sintchenko V, Gilbert GL. Rapid identification of methicillin-resistant *Staphylococcus aureus* transmission in hospitals by use of phage-derived open reading frame typing enhanced by multiplex PCR and reverse line blot assay. J Clin Microbiol. 2010;48(8): 2741–2748. doi: 10.1128/JCM.02201-09 20519463 PMC2916565

[57] O’Sullivan MV, Sintchenko V, Gilbert GL. Quantitative estimation of the stability of methicillin-resistant *Staphylococcus aureus* strain-typing systems by use of Kaplan-Meier survival analysis. J Clin Microbiol. 2013;51(1): 112–116. doi: 10.1128/JCM.01406-12 23100339 PMC3536198

[58] O’Sullivan MV, Zhou F, Sintchenko V, Gilbert GL. Prospective genotyping of hospital-acquired methicillin-resistant *Staphylococcus aureus* isolates by use of a novel, highly discriminatory binary typing system. J Clin Microbiol. 2012;50(11): 3513–3519. doi: 10.1128/JCM.01625-12 22895043 PMC3486244

